# Sperm DNA Methylation at Metabolism-Related Genes in Vegan Subjects

**DOI:** 10.3389/fendo.2021.633943

**Published:** 2021-03-09

**Authors:** Marica Franzago, Iva Sabovic, Sara Franchi, Maria De Santo, Andrea Di Nisio, Alice Luddi, Paola Piomboni, Ester Vitacolonna, Liborio Stuppia, Carlo Foresta

**Affiliations:** ^1^Department of Medicine and Aging, School of Medicine and Health Sciences, “G. D’Annunzio” University, Chieti-Pescara, Chieti, Italy; ^2^Center for Advanced Studies and Technology (CAST), “G. D’Annunzio” University, Chieti-Pescara, Chieti, Italy; ^3^Unit of Andrology and Reproductive Medicine, Department of Medicine, University of Padova, Padova, Italy; ^4^Department of Clinical and Experimental Sciences, University of Brescia, Brescia, Italy; ^5^Department of Psychological, Health and Territorial Sciences, School of Medicine and Health Sciences, “G. D’Annunzio” University, Chieti-Pescara, Chieti, Italy; ^6^Casa di Cura Privata Spatocco, Chieti, Italy; ^7^Department of Molecular and Developmental Medicine, University of Siena, Siena, Italy

**Keywords:** sperm, vegan, epigenetic, reproduction, nutrition

## Abstract

**Objective:**

To investigate if epigenome of sperm cells could be dynamically affected by nutrition.

**Design and Methods:**

We assessed 40 healthy volunteers with different dietary habits and collected their demographic characteristics, as well as clinical and anthropometric parameters. We compared methylation profiles in sperm quantified by bisulfite pyrosequencing, at promoter-associated CpG sites of genes involved in metabolism including fat mass and obesity-associated (*FTO*) and melanocortin-4 receptor (*MC4R*) from six vegans and 34 omnivores. In addition, the *FTO* rs9939609 (T>A) was genotyped.

**Results:**

Higher DNA methylation levels were detected in the sperm of vegan at *FTO* gene CpG1 (p=0.02), CpG2 (p=0.001), CpG3 (p=0.004), and CpG4 (p=0.003) sites and at *MC4R*-CpG2 site [p=0.016] as compared to sperm of omnivores. This association was not related to *FTO* genotype.

**Conclusions:**

Although limited by the small number of investigated cases, our data provide insight into the role of diet on sperm DNA methylation in genes involved in metabolism.

## Introduction

Epigenetic modifications, including DNA methylation, histone marks and small non-coding RNAs, are stable and mitotically heritable changes that modulate normal and disease-related phenotypic differences. Among environmental factors able to induce epigenetic modifications, a key role is played by diet. Dietary habits can result in epigenetic modifications by acting directly on metabolism genes to up- or downregulate pathways involved in the bioavailability of nutrients (1). In addition, it has been also suggested that nutrition induced epigenetic modifications of gene expression can influence metabolism and susceptibility to non-communicable diseases (NCDs) (2–4).

To date, growing evidence suggests that the sperm epigenome can be dynamically affected by lifestyle (nutrient supply, physical exercise, alcohol and tobacco consumption) influencing not only male individual health status, but also his reproductive fitness and even the future offspring’s health (5–9). Several studies have demonstrated that the spermatozoa from obese humans as well as from rats exposed to different models of diet, such as high-fat or low-protein diet, show an altered epigenetic signature, raising the question of a possible epigenetic inheritance of metabolic dysfunction (8, 10). Therefore, the possible role of diet in improving the quality of human sperm is becoming widely explored in the context of male infertility focusing on sperm epigenetic modifications (11).

Unlike omnivore diet, defined as a diet consuming all types of foods, the vegan diet is characterized by total exclusion of any animal derived substance, being thus very rich in fibres, but poor in proteins and fats (12). To date, this kind of diet is increasingly widespread in western societies, but its prevalence remains low (13). Although few studies described vegan diet as healthy, no conclusive data have been obtained yet (14–16), and vegan diet often requires supplementation of additional nutrients, at least during pregnancy (17).

To the best of our knowledge, the association between vegan diet and sperm DNA methylation patterns in human has not been investigated yet. In order to fill this gap, therefore, in the present study we investigated the effects of vegan diet as compared to omnivore diet on DNA methylation profiles in sperm at promoter-associated CpG sites of genes involved in metabolism, namely fat mass and obesity-associated (*FTO*) and melanocortin-4 receptor (*MC4R*).

*FTO* gene encodes for an AlkB-like 2-oxoglutarate-dependent nucleic acid demethylase, a potential regulator of RNA modification. It is highly expressed in the hypothalamus, visceral fat and liver but its function remains undefined. Recent studies reported that *FTO* seems to influence the Iroquois homeobox 3 (IRX3) expression with effects on body weight (18).

MC4R protein is a membrane-bound G-protein-coupled receptor found in brain regions, including the paraventricular nucleus in the hypothalamus (19) and common genetic variations near *MC4R* are involved in food intake by participating in appetite control and energy balance regulation (20–26).

Polymorphisms in these genes have been associated with body weight and composition, obesity, Type 2 Diabetes Mellitus (T2DM) and eating behavior (27–37).

The aim of this study is to evaluate the effect of vegan diet on sperm DNA methylation as a model to understanding the epigenetic effect of nutrition on male gametes and provide information for possible diet-based therapeutic strategies for improve male reproductive fitness.

## Materials and Methods

### Study Participants

This is a multicentric study involving the Unit of Andrology and Reproductive Medicine of the Department of Medicine of the University of Padua (Italy), the Laboratory of Molecular Genetics, School of Medicine and Health Sciences, “G. D’Annunzio” University of Chieti (Italy), the Center for Diagnosis and Cure of Couple infertility, University of Siena (Italy) and the Spatocco Clinic, Chieti (Italy). Forty healthy subjects with different dietary habits were recruited. In particular, six cases have been following a vegan diet for at least 2 years, and the remaining 34 an omnivore diet. Exclusion criteria were obstructive azoospermia and known causes of infertility (previous or concomitant testicular cancer, orchitis, testicular torsion and trauma, use of gonadotoxic drugs, oncological diseases, karyotype anomalies, Y chromosome long arm microdeletions, mutations in the androgen receptor and varicocele).

The study of sperm DNA methylation was conducted at the Laboratory of Molecular Genetics, School of Medicine and Health Sciences, “G. D’Annunzio” University of Chieti, Italy.

### Patients

During the visit, data on demographic characteristics, anthropometric, and clinical parameters were collected. In addition, smoking habits and supplement intake were reported. Finally, physical activity (PA) was assessed registering the different levels of intensity (low, moderate, and high PA). All subjects underwent semen donation by masturbation into sterile containers after 2–5 days of sexual abstinence. Samples were allowed to liquefy for 30 min and were examined for sperm count, viability, motility and morphology according to the WHO criteria (38).

Sperm were isolated by using Percoll gradient centrifugation. After decondensation with proteinase K for total of 4 h DNA was extracted using the QIAamp^®^ DNA Mini Kit according to manufacturer’s recommendations.

The study was approved by the Ethics Committee of the University of Padua, Italy (protocol number #2208). In accordance with the Declaration of Helsinki, all participants gave their written informed consent prior to their inclusion in the study.

### DNA Methylation Analysis

The promoter associated CpG sites were tested in *FTO* gene (16q12.2, 4 CpGs sites), *MC4R* gene (18q21.32, 2 CpGs sites) and the imprinted gene *H19* (11p15.5, 5 CpGs sites). *FTO* and *MC4R* genes were selected due to their correlation with obesity, metabolism and appetite control. *H19* gene was included as a control, being maternally expressed only and thus expected to show a full methylation in male gametes. After bisulfite treatment, DNA (~20 ng) was amplified by PCR using the Kapa Hifi Hotstart Uracil+ HotStart Ready mix (Roche Diagnostics), according to manufacturer’s recommendations.

Once NaBis-DNA amplified, the pyrosequencing was carried out using the PyroMark Q96 MD pyrosequencing instrument (Qiagen) with PCR and sequencing primers for the region of interest in the *FTO* and *MC4R* genes selected according to previous studies (39). In addition, PCR and sequencing primers for analysis of the *H19* CpGs were designed with PyroMark Assay Design (version 2.0.1.15; Qiagen). Primers information for these genes can be found in **Table 1**.

**Table 1:** Primers for bisulfite PCR and pyrosequencing.

Gene	Primers	Product size (pb)	Number of CpGs analyzed
***FTO***	F: TTTGGAGTTATTTTTTTTTTGAGTAGAAAR: [Btn]ATTCTCCTTAAACTCTAACCTATTTACTS: TTTTAGGTTAGATAGTTGGAAGA	168	4
***MC4R***	F: AGGGTGATATAGATTTAGATGTAGAAGTR: [Btn]AAACAATATACTTTCCATTTCATTTTACACS: GTAGAAGTTTTTGAAGTTTG	220	2
***H19***	F: GGTTTTGGAGGTTAGTGTTTTR: [Btn]CTCAACCCCTAAAACTAACTTAACAS: TTGTATTATTTTTTTTTTTGAGAGT	322	5

### Genotyping

The rs9939609 (T>A) SNP in *FTO* was genotyped in twenty-five subjects by PCR amplification (95°C for 10 min, followed by 35 cycles of 95°C for 30 s, 60°C for 30 s, 72°C for 30 s and a final extension at 72°C for 10 min) and direct sequencing procedure using BigDye Term v3.1 CycleSeq Kit (Life Technologies, Monza, Italy) followed by automatic sequencing analysis.

### Statistical Analysis

The subjects were stratified according to dietary habits and differences between the two groups were tested by the Mann-Whitney U for continuous variables or the Chi-square test for categorical variables. Shapiro-Wilk test showed a non-normal distribution of methylation levels. Thus, non-parametric analysis was conducted.

Moreover, to test the effect of genotypes on body mass index (BMI) as well as the effect of genotypes on methylation levels, a Nonparametric Kruskal-Wallis test was performed.

The quantitative variables were summarized as means and standard deviation (SD). Qualitative variables were summarized as percentage. All tests were 2-sided, and a level of statistical significance was set at p < 0.05. All analyses were performed with SPSS version 20.

## Results

The demographic and clinical characteristics of study participants are summarised in **Table 2**. No difference was present in mean age and in BMI between vegan and omnivorous men.

**Table 2:** Demographic and clinical characteristics of healthy vegans and omnivore.

Characteristics	omnivorous men (n=34)	Vegan (n=6)	p-value*
Age (yr)^a^	35.5 ± 7.07	36.3 ± 7.58	*0.86*
BMI (kg/m^2^)^a^	24.5 ± 2.47	25.7 ± 2.8	*0.19*
Smoking^b^			*0.34*
No	63.0	83.3	
Yes	37.0	16.7	
Physical activity^b^			*0.63*
Low	51.9	66.7	
Moderate	37.0	16.7	
High	11.1	16.7	
Sperm concentration^a^ (10^6/^ml)	79.7 ± 56.9	44.3 ± 21.2	*0.14*
Progressive Sperm motility^b^	57.2 ± 11.6	46.4 ± 22.4	*0.29*
Total Sperm motility^b^	59.2 ± 11.8	59 ± 23.7	*0.60*
Sperm morphology (normal forms)^b^	9 ± 3.3	10.2 ± 4.9	*0.64*

*Mann–Whitney U Test or Chi-square test.

^a^Data are presented as means ± SD.

^b^Data are presented as percent (%).

The vegans reported no assumption of any type of dietary supplement. In addition, no significant difference was reported in physical activity and smoking between the subjects in the two groups. Sperm count parameters showed no significant difference in the two groups. All subjects had a normal sperm count, except one subject in the omnivore group and one in the vegan group who had mild oligozoospermia (<15 million/ml) and severe oligozoospermia (<5 million/ml), respectively.

Methylation profiles of sperm DNA from all men recruited are reported in **Table 3**. Vegan subjects showed higher sperm DNA methylation levels as compared to omnivorous men at *FTO* gene CpG1 (p=0.02), CpG2 (p=0.001), CpG3 (p=0.004), and CpG4 (p=0.003) sites. In addition, the mean DNA methylation percentages for the overall CpGs at *FTO* gene in sperm of vegan men were higher than in sperm of omnivorous men (p=0.001).

**Table 3:** Methylation profiles in sperm from vegans and omnivore.

CpG Sites	omnivorous men (n=34)	Vegan (n=6)	p-value*
***FTO* gene**			
CpG1%	1.44 ± 1.11	2.83 ± 1.33	***0.024***
CpG2%	2.53 ± 2.11	7.0 ± 2.28	***0.001***
CpG3%	3.65 ± 2.45	7.83 ± 4.75	***0.004***
CpG4%	0.85 ± 1.02	2.33 ± 0.82	***0.003***
Mean all CpG sites %	2.06 ± 1.48	5.0 ± 2.19	***0.001***
			
***MC4R* gene**			
CPG1%	4.12 ± 1.53	4.17 ± 1.17	*0.80*
CpG2%	7.0 ± 2.26	10.17 ± 3.31	***0.016***
Mean all CpG sites %	5.65 ± 1.76	7.0 ± 2.28	*0.17*
***H19* gene**			
CpG1%	91.5 ± 2.63	90.5 ± 1.38	*0.23*
CpG2%	94.41 ± 2.66	96.33 ± 3.14	*0.17*
CpG3%	92.26 ± 2.38	95.17 ± 1.60	***0.007***
CpG4%	92.38 ± 3.95	95.33 ± 4.08	*0.11*
CpG5%	98.32 ± 2.52	100 ± 0	*0.05*
Mean all CpG sites %	93.74 ± 2.06	95.33 ± 1.86	*0.12*

*Mann–Whitney U Test.

Data are presented as means ± SD.

Statistically significant values are in bold.

DNA methylation levels at *MC4R*-CpG1 site were not significantly different between vegan and omnivorous men, whereas DNA methylation levels at *MC4R*-CpG2 site were significantly different between the two groups (p=0.016). On the other hand, all the analyzed CpG sites of the *H19* gene were close to 100% of methylation in all the samples, consistently with their maternal imprinting. However, DNA methylation levels at *H19* gene CpG sites were slightly lower in sperm DNA of omnivores than in vegan men, although only CpG3 value did reach significance (p=0.007).

No significant association was found for *FTO* genotype neither with BMI (**Supplementary Table 1**) nor with DNA methylation levels at *FTO* gene (**Supplementary Table 2**).

## Discussion

The epigenetics of human sperm currently represents a very promising field of investigation in relation to the growing evidence of the role played by epigenetic alterations in male infertility. Very recently, the presence of sperm epigenomic alterations has been used as a biomarker of the success rate of ART protocols, since patients with specific pattern of sperm DNA methylation are characterized by a poor outcome of these protocols (40). Given the reversible nature of epigenetic modifications, males with aberrant sperm DNA methylation can be considered as excellent candidates to investigate treatments aimed to induce a reversibility of epigenetic alterations. It has been suggested that personalized nutrition could prevent or reverse the detrimental epigenetic modifications induced by unhealthy lifestyles. Since the spermatogenesis process is completed in a few weeks, it could be suggested that a few months period of personal lifestyle care could restore a physiological epigenetic mark of the sperm (10, 41). In this view, a crucial point is represented by the knowledge of the specific effect of different diets on the epigenetic pattern of human sperm.

Despite the exact relationship between diet and the epigenome remains unclear, several studies reported an association between epigenetic modifications and dietary depletion or supplementation (1, 42–48).

Recent study suggests substantial differences in methylation of CpG sites and genes, particularly in regulatory regions, between vegans and non-vegetarians, laying the foundation for the identification of transcriptional alterations associated with diet-influenced methylation patterns (49). In addition, genome-wide methylation analysis in the DNA of blood cells showed epigenetic differences in methylation in vegans versus pescatarians and in vegans versus nonvegetarians. In particular, the authors found that vegans had a higher methylation in the majority of the differentially methylated sites, with DNA hypomethylation occurring in only 4% of all DM probes in the nonvegetarian comparison; this value was 33% for the pescatarian comparison (50).

Moreover, it has been demonstrated that the sperm epigenome may be responsive to dietary factors (41, 51). In this view, we investigated for the first time, to the best of our knowledge, DNA methylation profiles of metabolism-related genes and their associations with vegan diet in sperm of healthy subjects. Our results showed that a vegan diet was associated with higher sperm DNA methylation at the *FTO* and *MC4R* genes.

These findings seem to confirm that a specific diet can induce epigenetic modifications in human sperm. The mechanism underlying this process and the possible consequences must be analyzed in detail. A possible explanation is that hypermethylation of *FTO* and *MC4R* genes is the result of the increased number of dietary methyl donors available in vegan diet. In fact, nutrients include molecules that constitute DNA and histone methylation, such as methylfolate, choline, betaine, methionine, vitamins B12, B6, and B2 (52). Several studies showed the critical nutrients occurrence for each dietary regime (53–56). In particular, on the micronutrient level, one study carried out on the largest sample of vegan dieters worldwide reported that the men following a vegan diet have lower intake of saturated fatty acids (SFA), retinol, vitamin B12 and D, calcium, zinc than omnivorous diet. On the other hand, higher levels of magnesium, iron, folic acid, vitamin B1, C, and a higher intake of dietary fiber have been shown in vegan compared to omnivore dieters (57). The risk of nutrient deficiencies for specific micronutrients is the major criticism of plant-based diets. In fact, 4% of vegans are more likely to need supplements and food fortified compared to omnivores (13). Noteworthy, in the present study the vegans reported no kind of dietary supplement. Our findings suggest that the possible imbalances in the metabolism of the methyl nutrients such as vitamins (folate, riboflavin, vitamin B6, choline) and amino acids (methionine, cysteine, serine, glycine) could potentially modulate hypo- or hyper- methylation of DNA. An interesting point is the comparison between our results and those reported in the literature on obese subjects. In fact, it has been described that *FTO* and *MC4R* genes show different levels on DNA methylation in obese as compared to lean subjects (41). However, these authors analyzed different CpG islands in these genes and results of the different islands were not consistent. Nevertheless, these results confirm that *FTO* and *MC4R* methylation in human sperm can be affected by individual lifestyle. In our study, surprisingly, the vegans had a higher BMI than omnivores, although not statistically significant. This is in contrast with previous studies reporting lower BMI as compared to omnivores whose diet included more proteins and less fibres (58, 59).

Given the potential role of genotype of *FTO* on gene expression (6, 60), we analyzed the effect of genotypes on methylation levels. No significant association was found between genetic variant and DNA methylation levels at *FTO* gene. To note, rs9939609 was not evaluated in the studies of Doaei et al. (60, 61), which pointed to another *FTO* SNP.

This study has some limitations. First of all, the cross-sectional study design. Secondly, the main limit of the study is the small sample size. In the last years, the number of subjects who began to adopt a vegan dietary pattern has increased, but still limited to a minority. To confirm these data, an increase in the number of subjects enrolled and the representation of different dietary groups are needed. The literature on the effects of diet on epigenetic alterations in sperm is limited as studies in this field are hardly available, largely due to difficulties implied by the collection of samples. The possible role of a diet in improving the quality of human sperm is underexplored in the context of sperm epigenetic modifications. As matter of fact, the connection between a vegan diet and sperm DNA methylation patterns in human is widely unknown and needs further insight.

Thirdly, the assessment of diet was occurred by self-report. Fourthly, the examined genes do not play a role in sperm development and function.

A strength of this study is that, to our knowledge, this is the first attempt to evaluate the effects of vegan diet on DNA methylation level at genes involved in metabolism in sperm.

Interestingly, Soubry introduced the Paternal Origins of Health and Disease (POHaD) paradigm emphasizing the paternal transgenerational epigenetic inheritance of metabolic disorders (62). Therefore, the epigenetic modifications of spermatozoa can be transmitted to the offspring and subsequent generations, thus influencing their lifetime health. In this regard, it has been demonstrated that obesity is a factor able to induce reversible sperm epigenetic modifications (8), providing insight into a possible role of specific diets in improving the human sperm quality. In accordance with these observations, we suggest that genes related to metabolism could be susceptible to germ cell epigenetic modulation in response to nutritional status and diet. A greater understanding of epigenetic pattern as modifiable component in the periconceptional period may provide new findings and propose a novel conceptualization of susceptibility to metabolic disturbances. This domain of research is solid, but the knowledge of the underlying mechanism is currently still lacking.

## Data Availability Statement

The data underlying this article will be shared on reasonable request to the corresponding author.

## Ethics Statement

The studies involving human participants were reviewed and approved by Ethics Committee of the University of Padua, Italy protocol number #2208. The patients/participants provided their written informed consent to participate in this study.

## Author Contributions

The study was designed by CF and LS. CF, IS, PP, AL, and MS contributed to clinical evaluation and support to the recruitment of patients. IS, SF, and MF conducted the experiments. MF, IS, and LS contributed to data acquisition. MF, LS, and EV contributed to interpretation of results. MF performed the statistical analysis. The manuscript was drafted by MF and LS. All authors contributed to the article and approved the submitted version. LS and CF are the guarantors of this work.

## Conflict of Interest

The authors declare that the research was conducted in the absence of any commercial or financial relationships that could be construed as a potential conflict of interest.
