# Supplementary material for: Sperm DNA Methylation at Metabolism-Related Genes in Vegan Subjects
Source: Front Endocrinol (Lausanne). 2021 Mar 9;12:633943. doi: 10.3389/fendo.2021.633943 (PMC7985526; doi:10.3389/fendo.2021.633943)
Supplement: Supplementary file 1 [file Table_1.doc]

**Sperm DNA methylation at metabolism-related genes in vegan subjects**

*Marica Franzago^1,2^, Iva Sabovic^3,4^, Sara Franchi^2,5^, Maria De Santo^6^, Andrea Di Nisio^3^, Alice Luddi^7^, Paola Piomboni^7^, Ester Vitacolonna^1,2^, Liborio Stuppia^2,5^, Carlo Foresta^3^*

^1^Department of Medicine and Aging, School of Medicine and Health Sciences, “G. D’Annunzio” University, Chieti- Pescara, Via dei Vestini, 66100 Chieti, Italy

^2^Center for Advanced Studies and Technology (CAST), “G. D’Annunzio” University, Chieti-Pescara, Chieti, Italy

^3^Department of Medicine, Unit of Andrology and Reproductive Medicine, University of Padova, Italy

4Department of Clinical and Experimental Sciences, University of Brescia, Italy

^5^Department of Psychological, Health and Territorial Sciences, School of Medicine and Health Sciences, “G. D’Annunzio” University, Chieti-Pescara, Chieti, Italy

^6^Spatocco Clinic, Chieti (Italy)

^7^Department of Molecular and Developmental Medicine, University of Siena, 53100 Siena, Italy

**Supplementary Table 1- BMI of twenty-five participants according to genotypes (data are mean±SD).**

|  |  | |
| --- | --- | --- |
|  | **BMI** | **P*** |
|  |
| ***FTO* *rs9939609*** |  | 0.95 |
| ***TT (n=3)*** | 25.3±4.9 |
| ***AT (n=18)*** | 24.1±2.1 |
| ***AA (n=4)*** | 25.7± 4.7 |
| * Kruskall-Wallis | | |

**Supplementary Table 2- Methylation profiles in sperm of twenty-five participants according to genotypes (data are mean±SD).**

|  | **FTO CpG1 (%)** | **P*** | **FTO CpG2**  **(%)** | **P*** | **FTO CpG3**  **(%)** | **P*** | **FTO CpG4**  **(%)** | **P*** | **FTO mean**  **(%)** | **P*** |
| --- | --- | --- | --- | --- | --- | --- | --- | --- | --- | --- |
| ***FTO rs9939609*** |  | 0.95 |  | 0.22 |  | 0.79 |  | 0.99 |  |  |
| ***TT (n=3)***  ***AT (n=18)***  ***AA (n=4)*** | 1±0  1.33±1.37  1.25±0.96 |  | 1±0  2.83±2.28  1.50±1.92 |  | 1.67±1.53  4.17±2.36  2.25±1.71 |  | 0.67±0.58  0.83±1.04  0.75±0.96 |  | 1.33±0.58  2.22±1.59  1.50±1.29 | 0.52 |
|  |  |  |  |  |  |  |  |  |  |  |
|  |  |  |  |  |  |  |  |  |  |  |
| * Kruskall-Wallis | | | | | | | | |  |  |
